# Supplementary material for: Switched-capacitor-convertors based on fractal design for output power management of triboelectric nanogenerator
Source: Nat Commun. 2020 Apr 20;11:1883. doi: 10.1038/s41467-020-15373-y (PMC7171113; doi:10.1038/s41467-020-15373-y)
Supplement: Supplementary file 1 — Supplementary Information [file 41467_2020_15373_MOESM1_ESM.pdf]

Supplementary information

Switched-capacitor-convertors based on fractal design for output power management of triboelectric nanogenerator

Liu et al.

Supplementary Figure 1 | Comparison of Photographs and characteristics.

Supplementary Figure 2 | 8 units FSCCs with different orders.

Supplementary Figure 3 | Working mechanism and influence factors of 1st order FSCC.

Supplementary Figure 4 | Quantitative measurement for 1st order FSCC.

Supplementary Figure 5 | Quantitative measurement of voltage and output charge for FSCC.

Supplementary Figure 6 | Operating mechanism of 4=2×2 FSCC power management system.

Supplementary Figure 7 | Pulse output power management system and output of TENG .

Supplementary Figure 8 | output Charge of FSCC with different charge storage capacitors.

Supplementary Figure 9 | Circuit of 96=2×2×2×2×2×3 FSCC.

Supplementary Figure 10 | Photograph of 96=2×2×2×2×2×3 FSCC.

Supplementary Figure 11 | Input and output performance of 96=2×2×2×2×2×3 FSCC.

Supplementary Figure 12 | The output power of TENG with/without FSCC.

Supplementary Figure 13 | Preliminary research for pulse power output.

Supplementary Figure 14 | The voltage accumulated effect..

Supplementary Figure 15 | Pulse energy output of FSCC power management.

Supplementary Figure 16 | The measured current-time curve with 96=2×2×2×2×2×3 FSCC.

Supplementary Figure 17 | Lighting LEDs.

Supplementary Figure 18 | The driving current of electric devices .

Supplementary Figure 19 | The stability performance of 96=2×2×2×2×2×3 FSCC.

Supplementary Table 1 | Parameter comparison of traditional transformers and FSCC.

Supplementary Table 2 | Total output voltage drop of different units with 2nd order FSCC.

Supplementary Table 3 | Total output voltage drop of different orders with 24 units FSCC.

Supplementary Table 4 | Comparison of $V_{d,t}$ between 1st and Lth orders $\mathrm{N} = 2^L$ FSCC .

Supplementary Table 5 | Parameters of FSCCs (FSCCs are integrated on breadboard).

Supplementary Note 1 | Theoretical analysis of FSCC.

Supplementary Note 2 | Minimum total output voltage drop for FSCC

Supplementary Note 3 | Working principle of FSCC power management system for TENG.

Supplementary Note 4 | Preliminary research for maximum pulse power output.

Supplementary Note 5 | Total energy transfer efficiency under pulse output.

Supplementary Note 6 | Total energy transfer efficiency under constant output.

Supplementary Figures

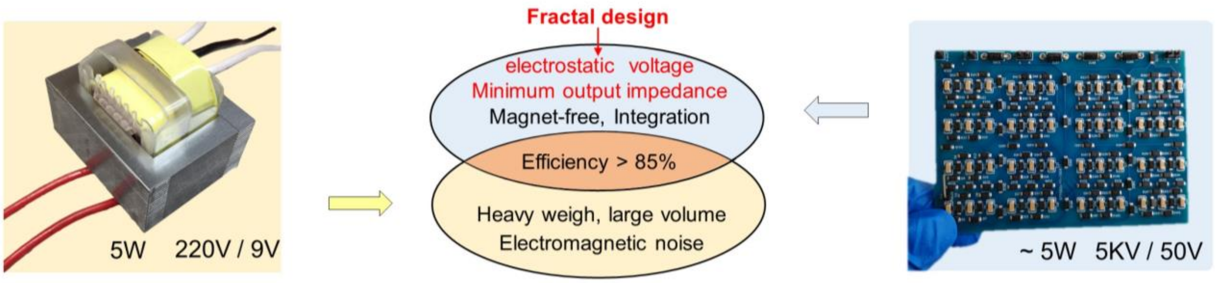

Supplementary Figure 1 | Comparison of Photographs and characteristics. A 42×40×35 mm^3 conventional step-down transformer and designed 4×60×85 mm^3 step-down FSCC (Consisting of diodes and capacitors and being used in this work).

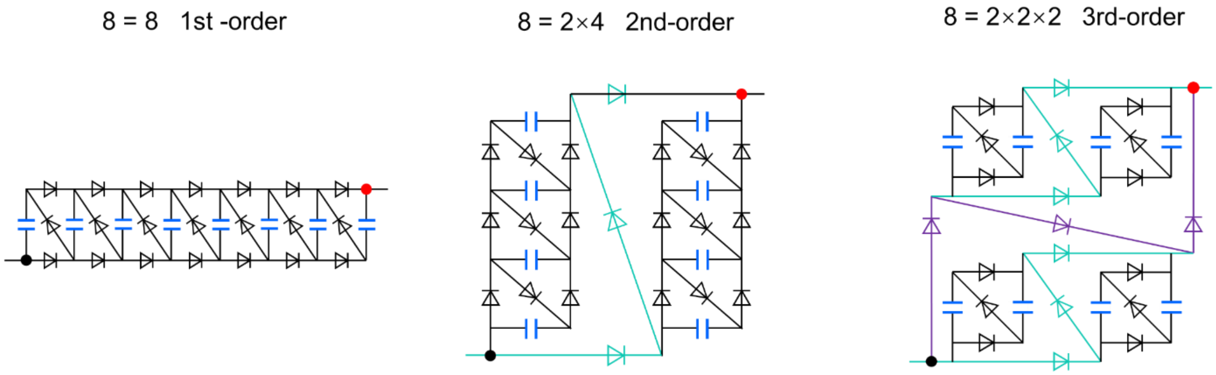

Supplementary Figure 2 | 8 units FSCCs with different orders.

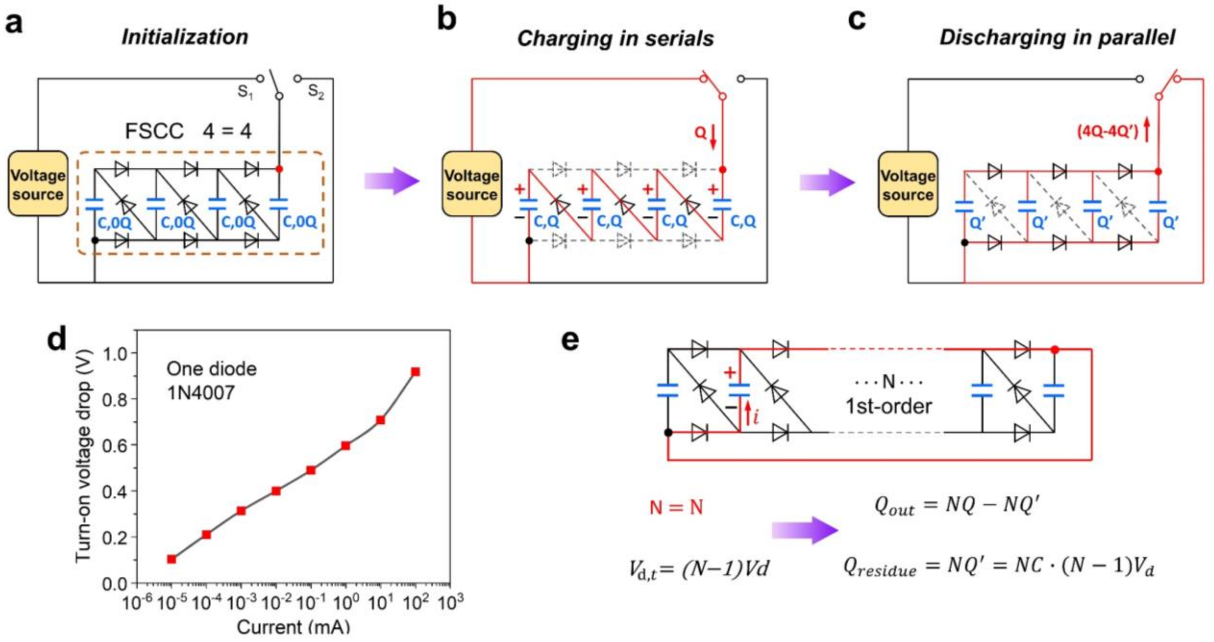

Supplementary Figure 3 | Working mechanism and influence factors of 1st order FSCC. a-c, The step-down process of 4=4 FSCC during a charging/discharging cycle, the FSCC can achieve multiple charge output through the auto-switch of capacitors from serial to parallel connection. **d**, The turn-on voltage drop of one diode with different currents. **e**, Theoretical analysis of the influence of total output voltage drop on 1st order FSCC.

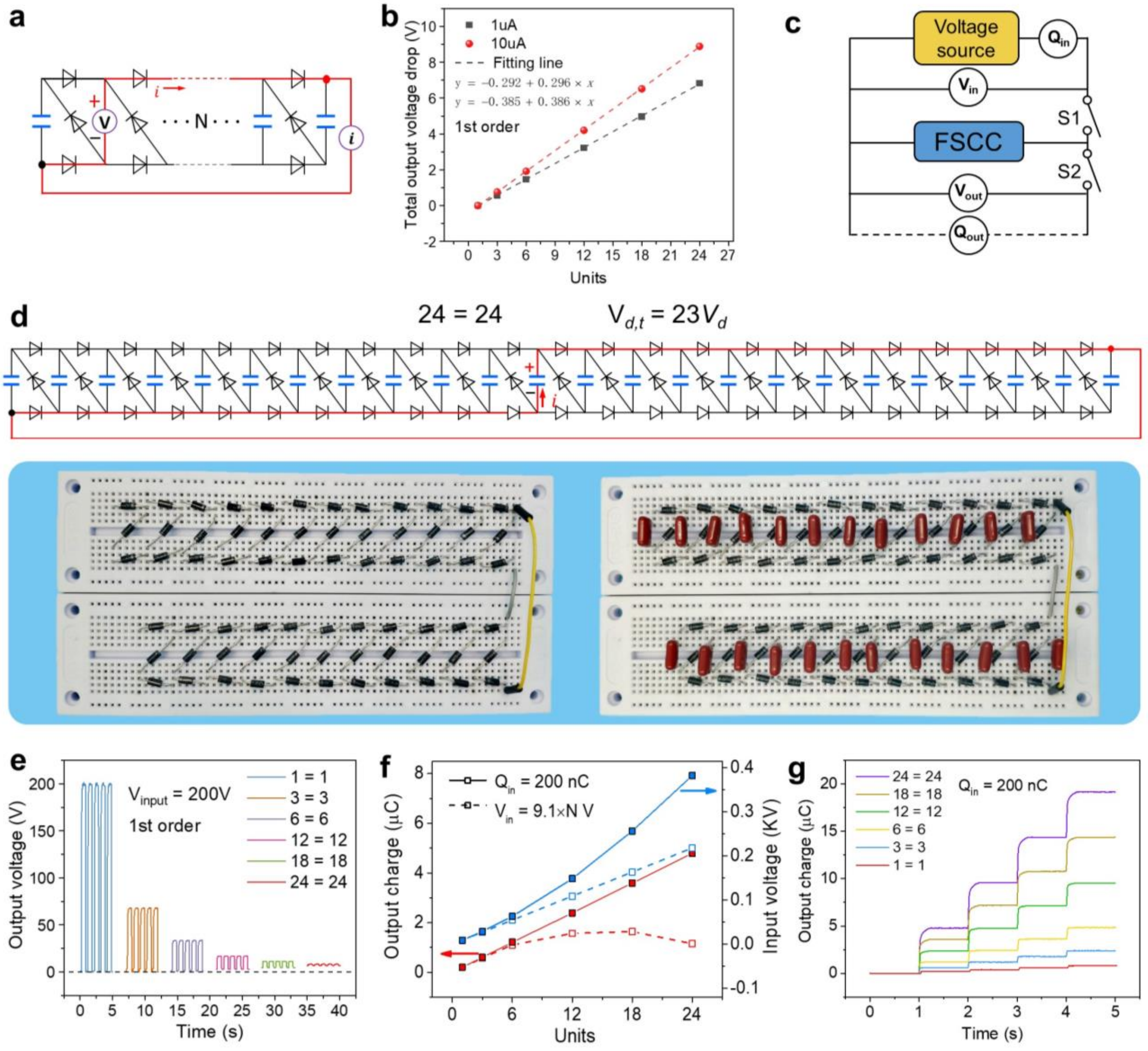

Supplementary Figure 4 | Quantitative measurement for 1st order FSCC. a, Circuit of testing turn-on voltage drop of FSCC, a voltage source is used while testing, and **b,** The corresponding total output voltage drop versus units curve. **c**, Schematic diagram of using voltage source to quantify FSCC output performance. **d,** Circuit of 1st order 24 units FSCC, photograph below it is corresponding testing circuit with charge storage capacitor (right) and without (left) (Integrated on breadboard). **e-f**, Output voltage and charge of 1st order FSCC with different units, the voltage drop has a significant influence on output voltage and charge. **g**, Output charge waveform with different units FSCC when input charge is 200 nC.

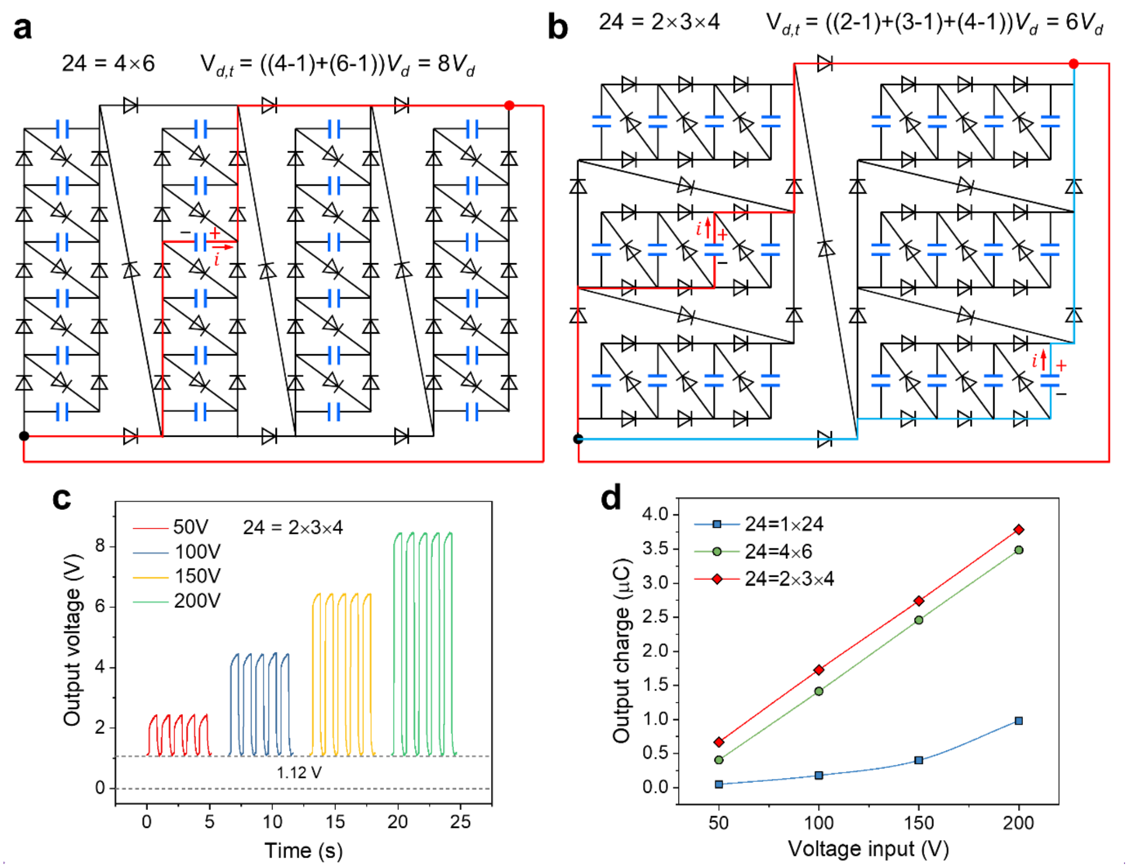

Supplementary Figure 5 | Quantitative measurement of voltage and output charge for FSCC. **a-b,** Circuit of 2nd order **a** and 3rd order **b** 24 units FSCC. **c**, Output voltage of 24=2×3×4 FSCC at different input voltages. **d**, Output charge of different orders FSCC at different input voltages.

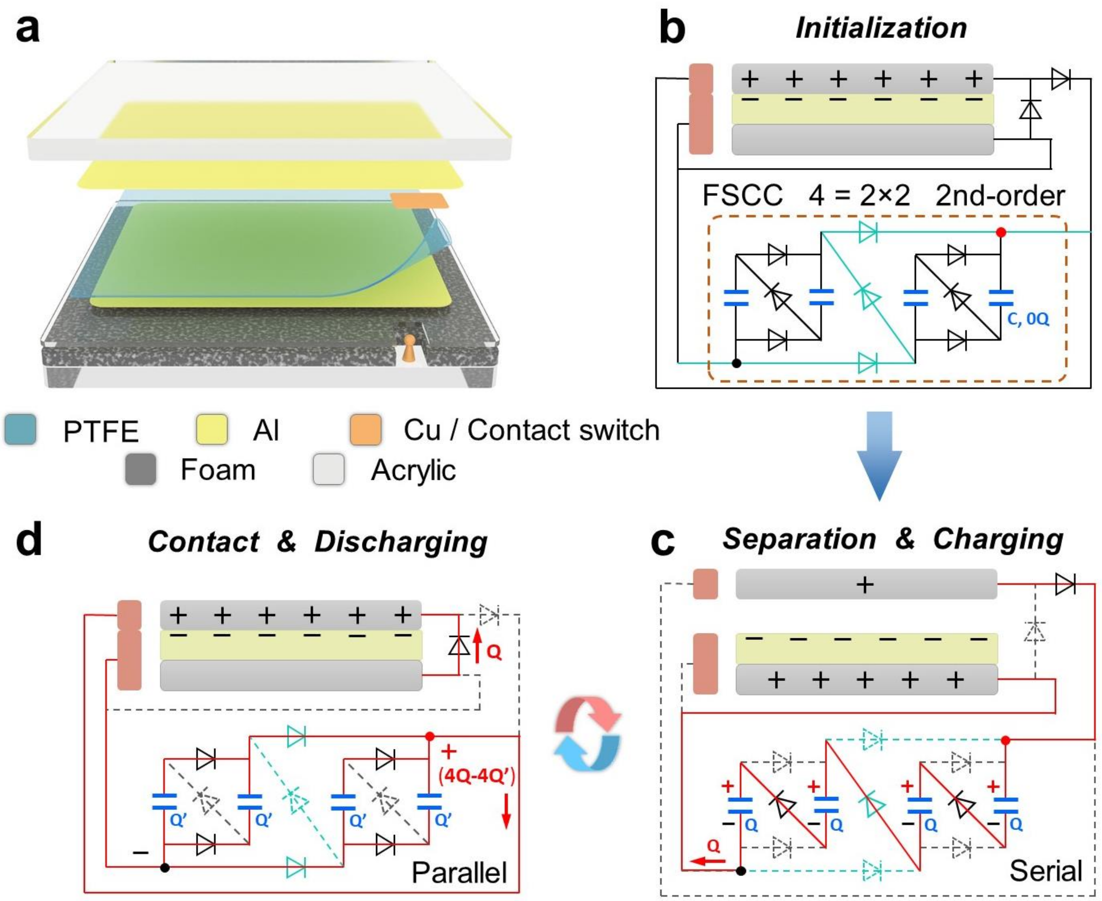

Supplementary Figure 6 | Operating mechanism of 4=2×2 FSCC power management system. **a,** The 3D illustration of TENG with a contact switch. **b-d,** The operating mechanism of 4=2×2 FSCC power management system for TENG during a contact-separation cycle.

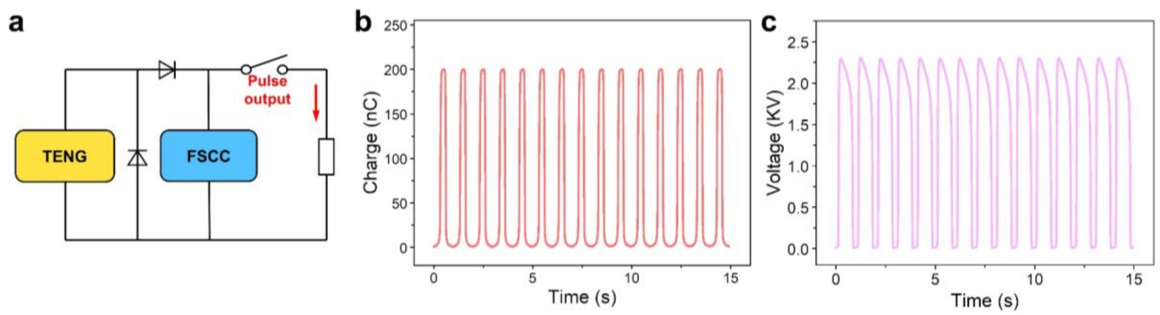

Supplementary Figure 7 | Pulse output power management system and output of TENG. a FSCC power management circuit with the pulse output. **b-c** Short-circuit charge and open-circuit voltage of TENG.

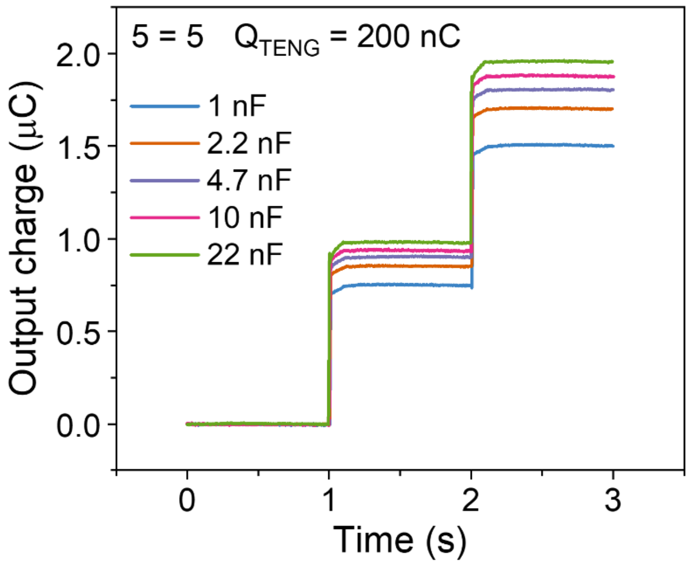

Supplementary Figure 8 | Output charge of FSCC with different charge storage capacitors.

$$96 = 2\times2\times2\times2\times2\times3$$

$$V_{d,t} = ((2\text{-}1)+(2\text{-}1)+(2\text{-}1)+(2\text{-}1)+(2\text{-}1)+(2\text{-}1)+(3\text{-}1))\cdot V_d = 7V_d$$

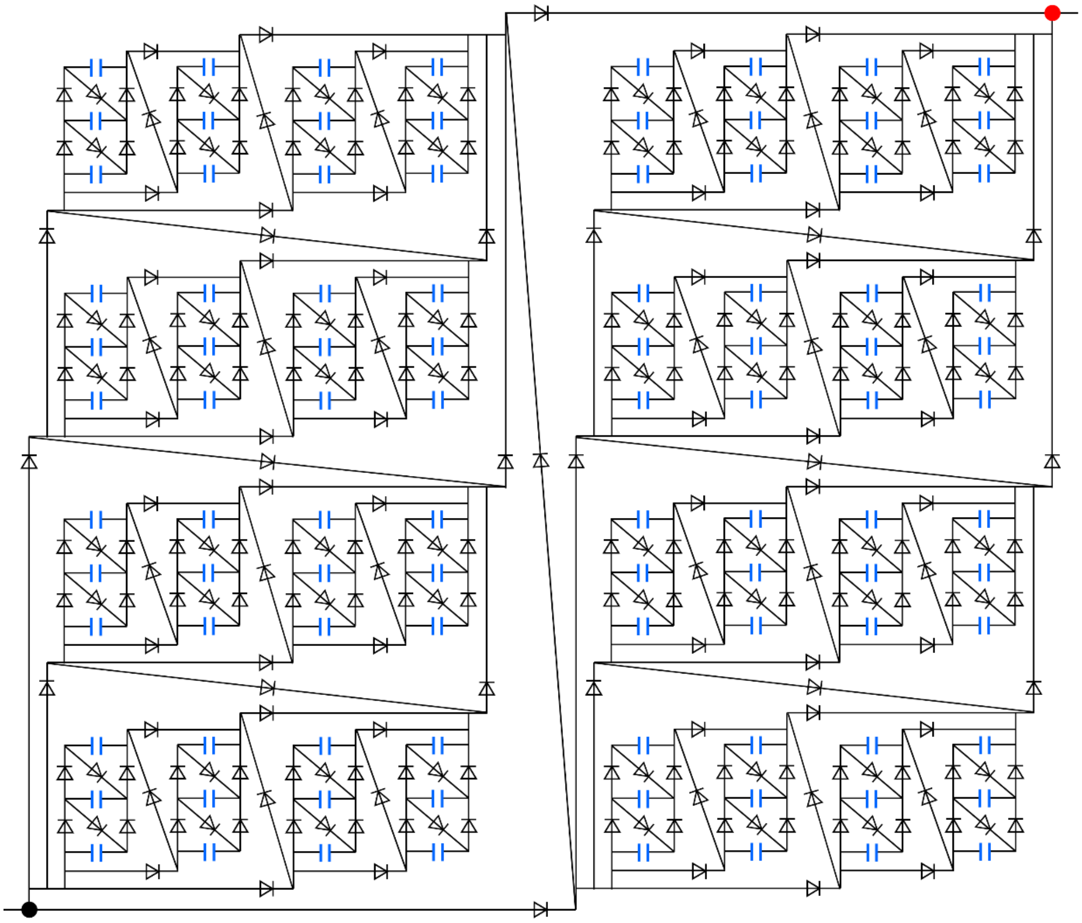

Supplementary Figure 9 | Circuit of 96=2×2×2×2×2×3 FSCC.

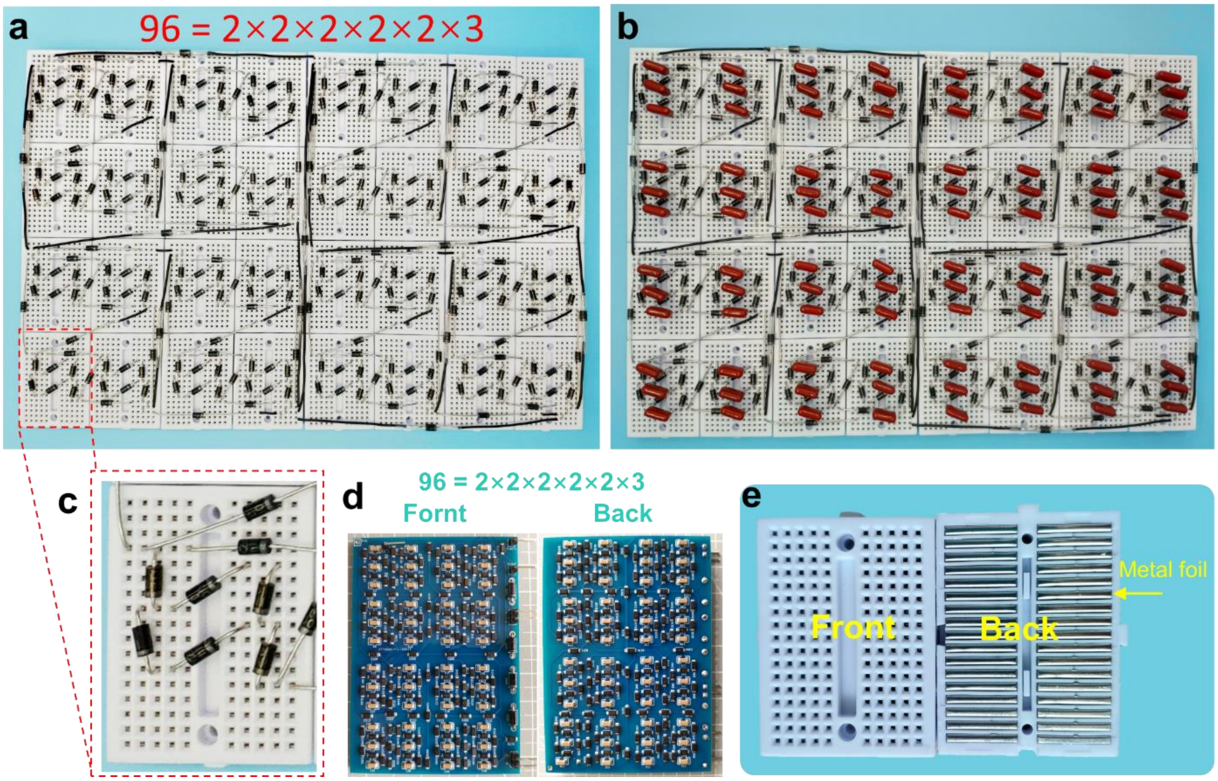

Supplementary Figure 10 | Photograph of 96=2×2×2×2×2×3 FSCC. a-b, FSCC Without/with charge storage capacitor, and **c** the detailed photo of the selected one unit that integrated on breadboard. **d**, FSCC integrated on PCB. **e**, Structure of the breadboard.

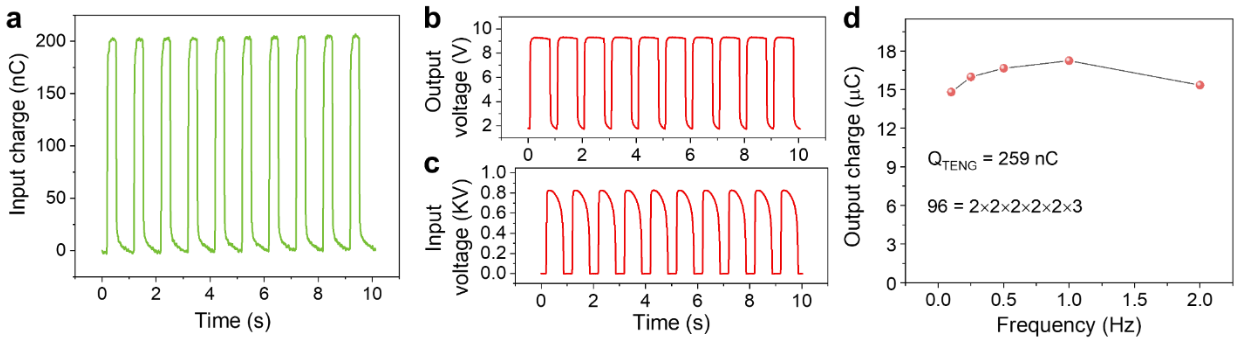

Supplementary Figure 11 | Input and output performance of 96=2×2×2×2×2×3 FSCC. a-c, The input charge, output voltage and input voltage. **d**, The output charge at different frequencies.

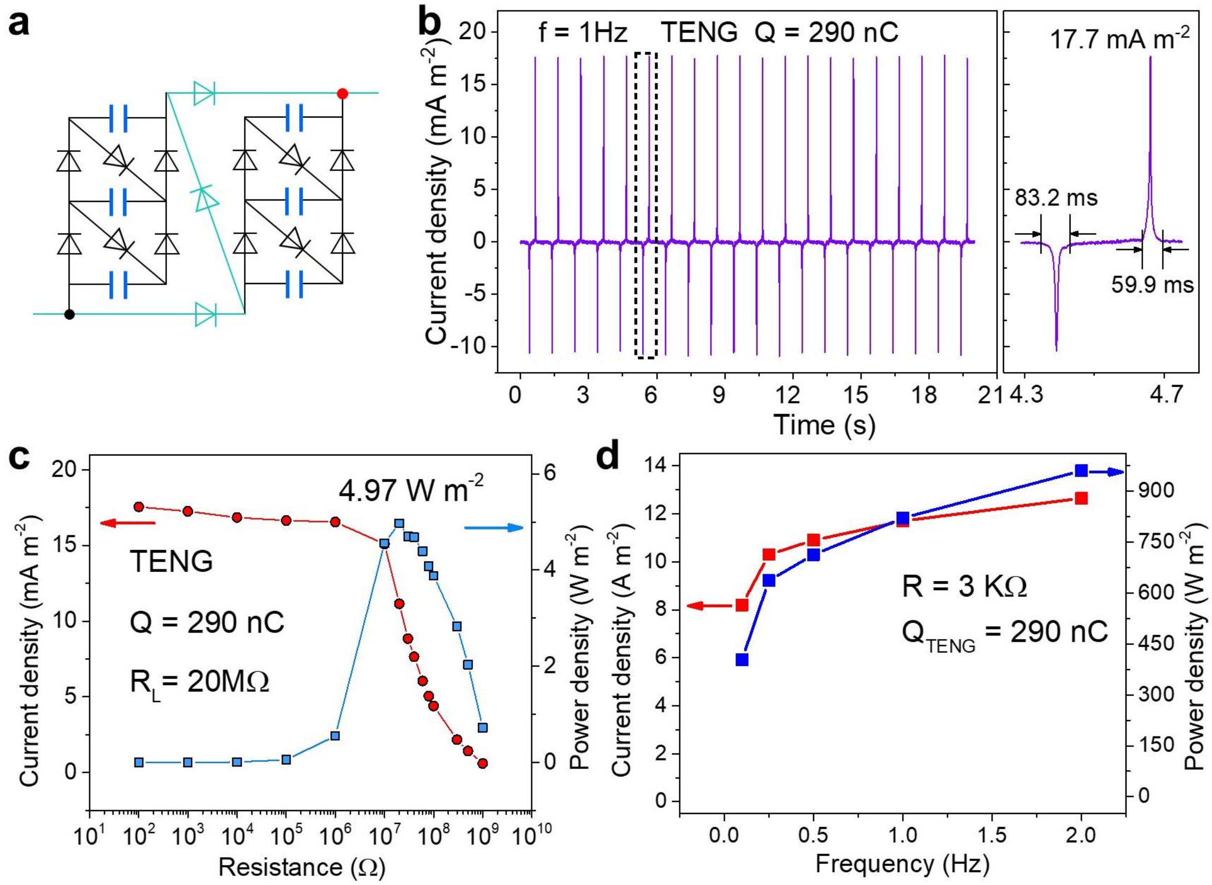

Supplementary Figure 12 | The output power of TENG with/without FSCC. a, Circuit of 6=2×3 FSCC. **b-c**, Short-circuit current and power density of TENG. **d**, Power output of 6=2×3 FSCC at different frequencies.

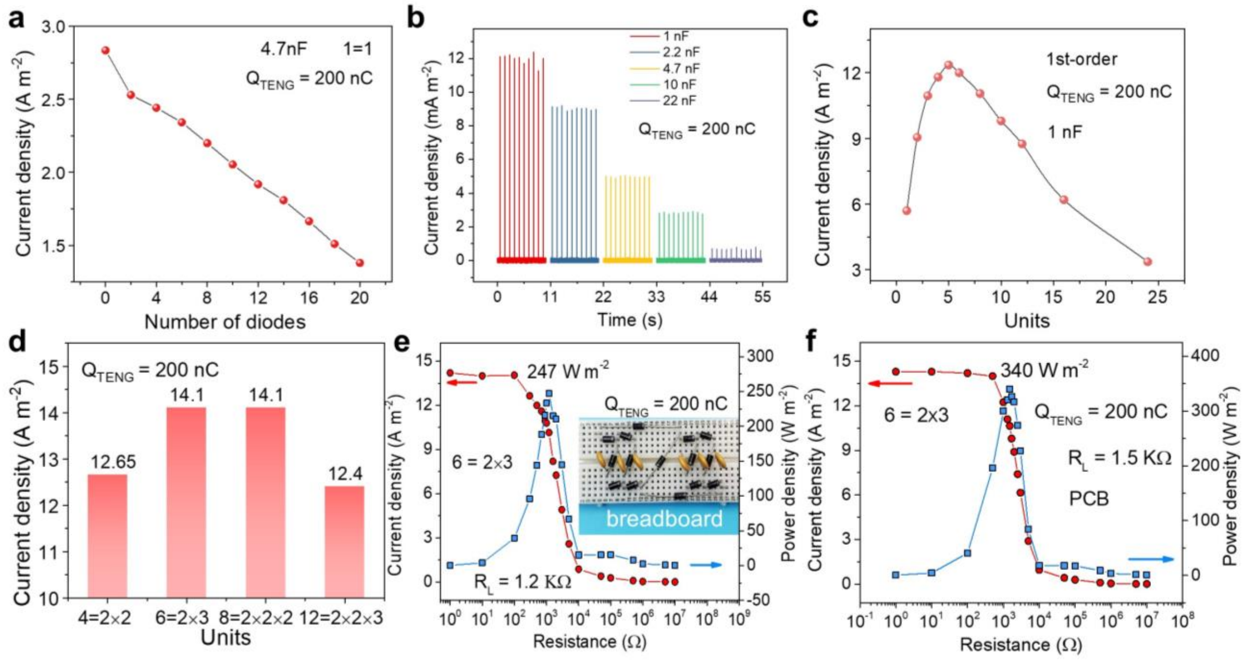

Supplementary Figure 13 | Preliminary research for pulse output power. a, Short-circuit current of 1=1 FSCC with different number diodes in series. **b,** The current density of 5=5 FSCC with different charge storage capacitor. **c,** The current output of 1st order FSCC with different units. **d**, The current output of FSCC with different units and orders. **e**, Power output of the power management system, the 6=2×3 FSCC is integrated on the breadboard. **f**, Power output of the power management system, FSCC is integrated on PCB.

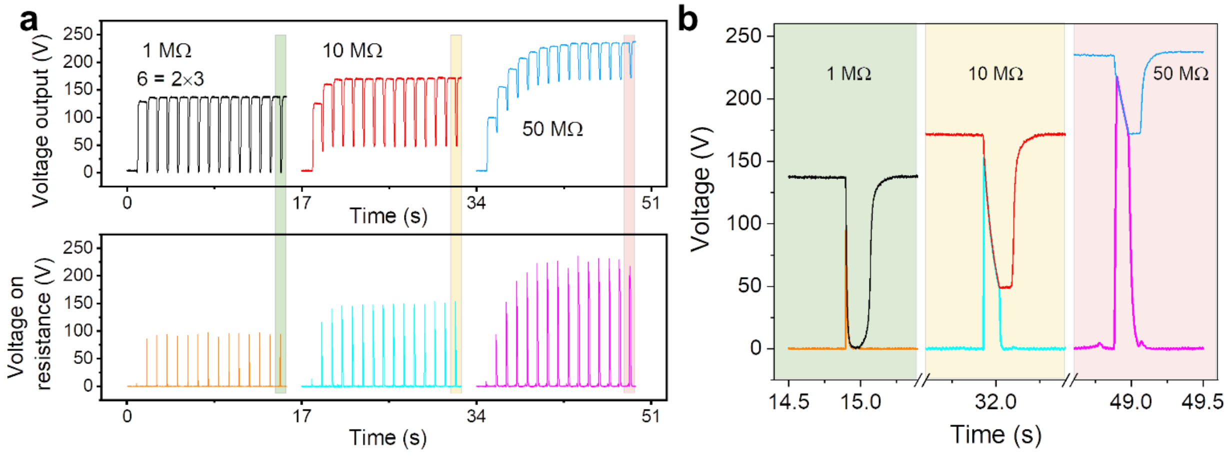

Supplementary Figure 14 | The voltage accumulated effect. Voltage output of 6=2×3 FSCC and voltage on the resistance under different loads **a**, and **b**, detailed waveform of the selected area.

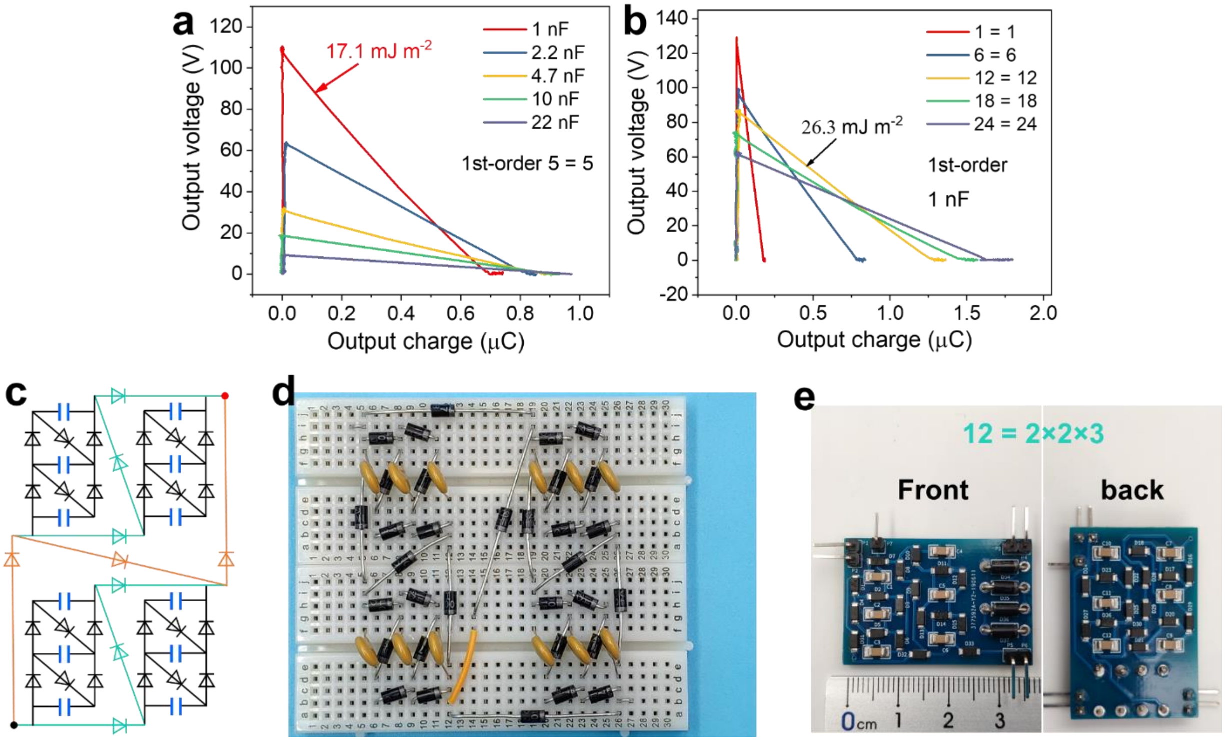

Supplementary Figure 15 | Pulse energy output of FSCC power management. a, Output energy of a 5=5 FSCC with different charge storage capacitors. **b**, Output energy of a 5=5 FSCC with different orders. **c**, The circuit of 12=2×2×3 FSCC. **d-e**, Photographs of 12=2×2×3 FSCC that integrated on breadboard **d** and PCB **e**.

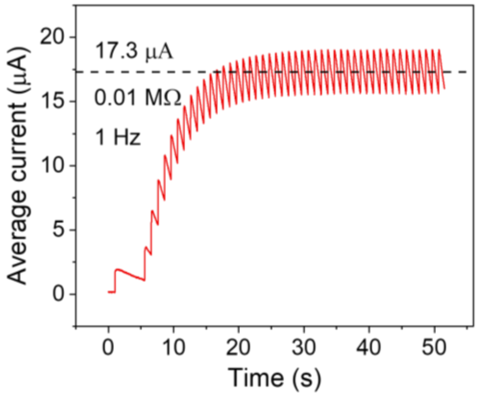

Supplementary Figure 16 | The measured current-time curve with 96=2×2×2×2×2×3 FSCC.

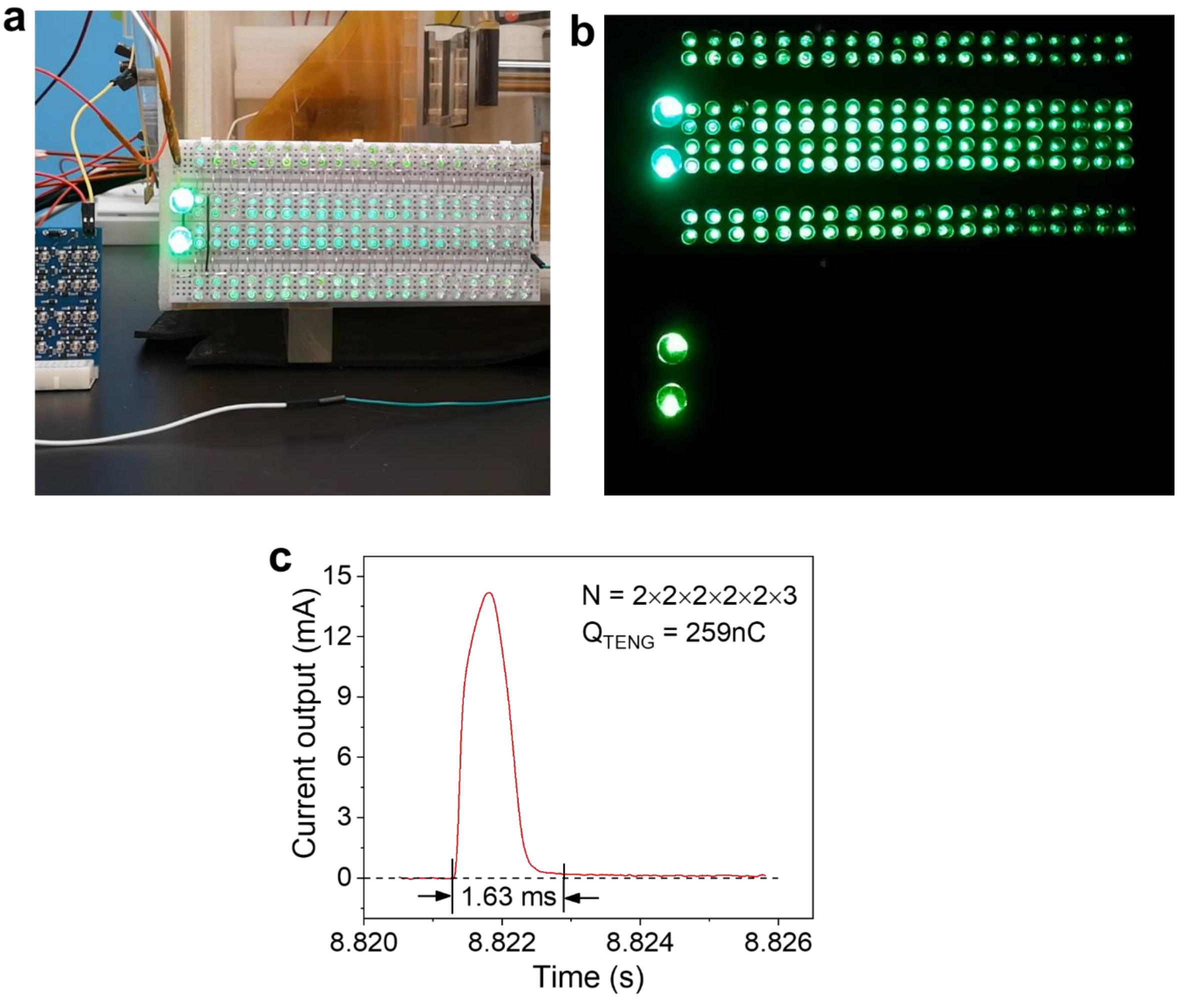

Supplementary Figure 17 | Lighting LEDs. a, Lighting the green LEDs in bright environment with 96=2×2×2×2×2×3 FSCC. **b,** Lighting green LEDs with 6=2×3 FSCC. **c**, Short-circuit current of 96=2×2×2×2×2×3 FSCC.

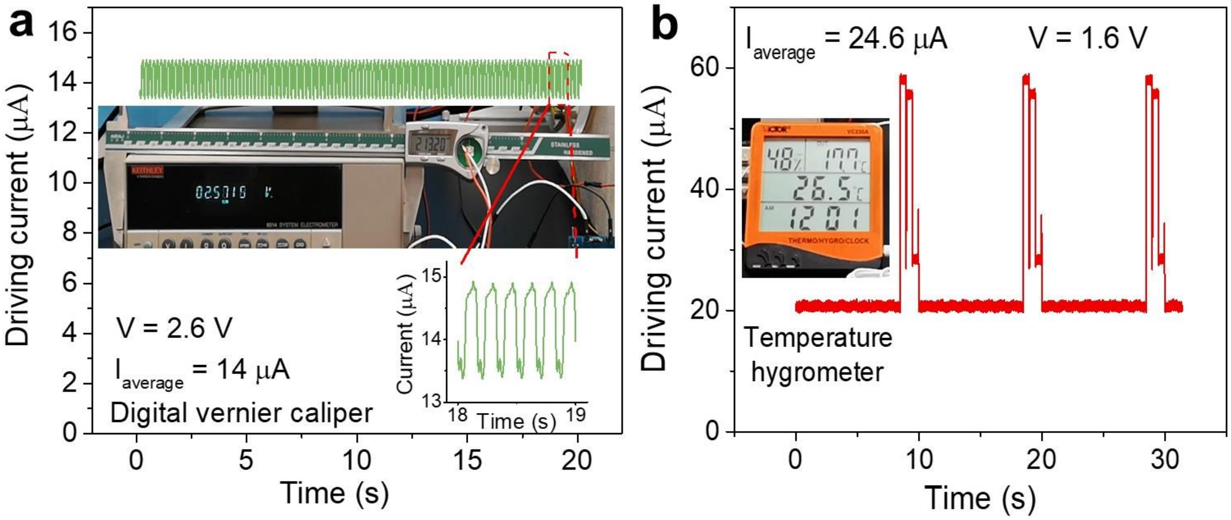

Supplementary Figure 18 | The driving current of electric devices. a Digital vernier caliper and **b** temperature hygrometer driven by a constant voltage source.

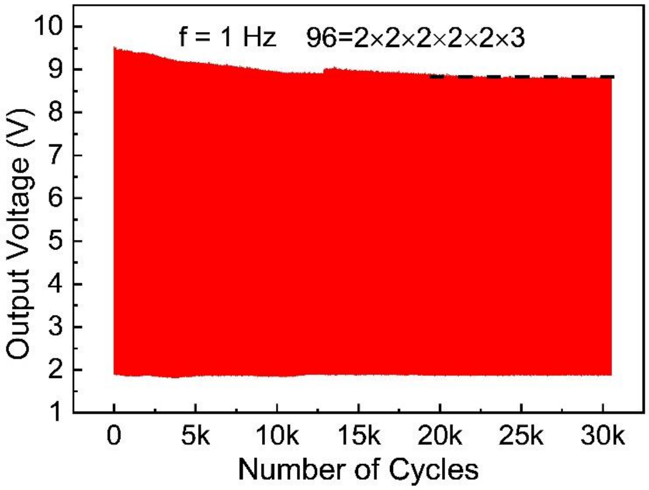

Supplementary Figure 19 | The stability performance of 96=2×2×2×2×2×3 FSCC.

Supplementary Tables

Supplementary Table 1 | Parameter comparison of traditional transformers and FSCC.

**Transformer**	**Rated power (w)**	**Size (mm)**	**Wight (g)**	**step-down ratio**	**Rated voltage (V)**	**Cost (RMB)**	**Efficiency**	**Applicable frequency**
Traditional transformer	5	42×40×35	208	24：1	220 : 9	>= 11	>90%	>= 50 Hz
FSCC	~5	85×60×4	25	96：1	5kV : 50	< 24	>90%	> 0.1 Hz

Supplementary Table 2 | Total output voltage drop of different units with 2nd order FSCC

N, L=2nd	$\sqrt[2]{N}$	$V_{d,t}$ (unit: V_d)			
16	4	4+4-2=6	2+8-2=8		
24	4.89	4+6-2=8	3+8-2=9	2+12-2=12	
64	8	8+8-2=14	16+4-2=18	32+2-2=32	
96	9.8	8+12-2=18	6+16-2=20	4+24-2=26	2+48-2=48

Supplementary Table 3 | Total output voltage drop of different orders with 24 units FSCC

N=24	$V_{d,t}$ (unit: V_d)	$\sqrt[L]{24}$	Expansion term		
L=1st		24	$1 \times 24 = 24$		
	$V_{d,t}$ (V_d)		23		
L=2nd		4.89	$4 \times 6 = 24$	$3 \times 8 = 24$	$2 \times 12 = 24$
	$V_{d,t}$ (V_d)		8	9	12
L=3th		2.88	$2 \times 3 \times 4 = 24$	$2 \times 2 \times 6 = 24$	
	$V_{d,t}$ (V_d)		6	7	
L=4th		2.21	$2 \times 2 \times 2 \times 3 = 24$		
	$V_{d,t}$ (V_d)		5		

Supplementary Table 4 | Comparison of $V_{d,t}$ between 1st and Lth orders $\mathrm{N} = 2^L$ FSCC

Units	1st order	Lth order		$V_{d,t}$ decrease times
	$V_{d,t}$ (unit: V_d)	Expansion term	$V_{d,t}$ (unit: V_d)	
2	1	2^1	1	0
4	3	2^2	2	0.5
8	7	2^3	3	1.3
16	15	2^4	4	2.75
32	31	2^5	5	5.2
64	63	2^6	6	9.5
128	127	2^7	7	17.1
256	255	2^8	8	30.9
512	511	2^9	9	55.8
1024	1023	2^{10}	10	100.3

Supplementary Table 5 | Parameters of FSCCs (FSCCs are integrated on breadboard).

	4 units	9 units	24 units	36 units	48 units	64 units	96 units
1st order	4=4	9=9	24=24				
2nd order	4=2×2	9=3×3	24=4×6	36=6×6	48=6×8	64=8×8	96=8×12
3rd order			24=2×3×4	36=3×3×4	48=3×4×4	64=4×4×4	96=4×4×6
6th order						64=2×2×2 ×2×2×2	96=2×2×2 ×2×2×3

Supplementary Notes

Supplementary Note 1 | Theoretical analysis of FSCC

Fig. 2a-c shows the operating principle of FSCC with charging in serial and discharging in parallel. For a N units FSCC, when voltage of V_{in} is applied to FSCC, every charge storage capacitor is charged to voltage of $V_{in}/\mathrm{N} - V_d$, considering the voltage drop of one diode V_d. When FSCC discharges, all charge storage capacitors are in parallel and the total output voltage drop $V_{d,t}$ would impede this process, therefore, the relationship of effective output voltage V_{out} and input voltage is as follow:

$$V_{out} = \frac{V_{in}}{N} - V_d - V_{d,t} \quad (1)$$

It is particularly important to note that the maximum value of measured voltage is not impeded by $V_{d,t}$ on the condition of open-circuit, because the current is zero under open circuit. And the measured open circuit voltage $V_{out,open}$ is as follow.

$$V_{out.open} = \frac{V_{in}}{N} - V_d \quad (2)$$

The measured open circuit voltage in Supplementary Equation 2 of FSCC, shown in Fig. 2e and Supplementary Figure 5c, is the sum of effective output voltage in Supplementary Equation 1 and total output voltage drop.

For the first charging/discharging cycle, the charge quantity in charge storage capacitors is zero, and charges of $V_{d,t} \cdot C_s$ will remain in charge storage capacitors while discharging. Hence, the relationship considering $V_{d,t}$ of output charge and input charge is as follow.

$$Q_{out,first} = N(Q_{in,first} - V_{d,t}C_s) \quad (3)$$

When the charging/discharging process of FSCC reaches to stable state after serval cycles, there is only $Q_{in,first} - V_{d,t}C_s$ charge being charged into FSCC, and $N(Q_{in,first} - V_{d,t}C_s)$ charge being released from FSCC while discharging. Hence, the relationship of input charge Q_{in} and output charge Q_{out} at stable state is described strictly by the following equation.

$$Q_{out} = NQ_{in} \quad (4)$$

The charge storage capacitors are all the same for consistency of output voltage. The charging process of FSCC is equal to charging capacitors in serial connection. Equation 5 gives relationship of Q_{in} and V_{in}.

$$Q_{in} = (V_{in} - NV_d + V_d) \cdot \frac{C_s}{N} \quad (5)$$

The discharging process is equal to capacitors discharge in parallel connection, and equation 6 gives relationship of Q_{out} and V_{out}.

$$Q_{out} = V_{out} \cdot C_s N \quad (6)$$

The energy conversion efficiency of FSCC η_{FSCC} is defined by the ratio of output energy to input energy of FSCC, it is used to describe the efficiency of FSCC itself only and belong to efficiency of part, and is given as follow:

$$\eta_{FSCC} = \frac{E_{out}}{E_{in}} = \frac{\int V_{out} dQ_{out}}{\oint V_{in} dQ_{in}} \quad (7)$$

Supplementary Note 2 | Minimum total output voltage drop for FSCC

The total output voltage drop $V_{d,t}$ of a 1st order N=m_1 FSCC is $(m_1 - 1)V_d$, and for L order N units FSCC, the total output voltage drop between every orders is in serial, which means that the total output voltage drop of L order FSCC is equal to the sum of output voltage drop of every order FSCC. Thus, the relationships of L, N and total output voltage drop $V_{d,t}$ are described by following equations:

$$N = \prod_{i=L}^{1} m_i \quad (8)$$

$$V_{d,t} = (\sum_{i=L}^{1} m_i - L) \cdot V_d \quad (9)$$

The minimum total output voltage drop $V_{d,t,min}$ can be described as follow:

$$V_{d,t,min} = (\sum_{i=L}^{1} m_i - L)_{min} \cdot V_d \quad (10)$$

To obtain $V_{d,t,min}$ of FSCC, best parameter of m_i is needed to solve by combining Supplementary Equation 8 and 9, that is, to solve the minimum sum of expanded factors of an integer, which is a very complex mathematical problems and is not the key point of this work. Fortunately, we can get the solution by enumeration method, the $V_{d,t}$ of FSCC with different orders and units is listed in Supplementary Table 2, 3, we can know that all m_i is closer to $\sqrt[L]{N}$ and $V_{d,t}$ is smaller, in other words, a judgment criterion to solve the $V_{d,t,min}$ is obtained as follow:

$$SUM_{min} = (\sum_{i=L}^{1}(m_i - \sqrt[L]{N}))_{min} \rightarrow V_{d,t,min} \qquad (SUM \geq 0) \quad (11)$$

Supplementary Note 3 | Working principle of FSCC power management system for TENG

To simplify the discussion, we chose a 4=2×2 FSCC to understand its power management mechanism. Supplementary Figure 6b shows the initialization state of FSCC power management system with electrodes in contact states. At this moment, positive charge is in the top electrode, the charge and capacitance of capacitors are 0Q and C, respectively. During the first charging/discharging process, the two electrodes are separated, and charge quantity of Q is charged into FSCC with charge storage capacitors in series (Red route in Supplementary Figure 6c). Therefore, charge quantity of Q is obtained in every charge storage capacitor. When the electrode gets into contact with switch contact again, the switch turns on, causing the charge storage capacitors to turn on. Automatically, the charge storage capacitors discharge (Red route in Supplementary Figure 6d) in parallel connection state due to the unidirectional conductivity of diode, and charge quantity of $4\mathrm{Q} - 4\mathrm{Q}'$ is released, with Q' remained in each capacitor. A stable state is reached after the first cycle, and there is only $\mathrm{Q} - \mathrm{Q}'$ charge being charged into FSCC and $4\mathrm{Q} - 4\mathrm{Q}'$ charge being released.

Supplementary Note 4 | Preliminary research for maximum pulse power output.

The discharging process of FSCC is equivalent to discharging process of a big capacitor, can be described by equation below:

$$i = \frac{U_0}{R} e^{-\frac{t}{RC}} \tag{12}$$

Where C and U_0 is respectively the capacitance and the voltage of capacitor. So the discharging process of FSCC can be conducted according to Supplementary Equation 4 ($Q_{out} = NQ_{in}$) and 6 ($Q_{out} = V_{out} \cdot C_s N$).

$$i = \frac{Q_{in}}{RC_s} e^{-\frac{t}{RNC_s}} \tag{13}$$

In the equation above, as $e^{-\frac{t}{RNC_s}} \in (1\sim0)$ is a decay factor, the maximum value of current is:

$$i_{max} = \frac{Q_{in}}{RC_s} \tag{14}$$

Therefore, that the maximum current is inversely proportional to the capacitance of charge storage capacitor.

Preliminary researches are carried out with TENG of 200 nC charge quantity for quantitative characterization. Firstly, the influence of voltage drop is discussed in Supplementary Figure 13a, and total voltage drop could reduce output current linearly. Supplementary Figure 13b shows that small (1 nF) charge storage capacitor is suitable to obtain high current in experimental aspect, which is consistent to theoretical analysis. For the influence of voltage drop. The current output under different units indicates that TENG with 5 units FSCC has a maximum current output (Supplementary Figure 13c). Supplementary Figure 13d deeply discusses the impacts of orders on

current, and shows that 6=2×3 and 8=2×2×2 FSCC can realize maximum output current. At last, we chose 6=2×3 FSCC for considering its smaller size in experiment. Finally, when FSCC is integrated on breadboard and on PCB (Supplementary Figure 13e-f), the maximum pulse power reaches 247 W m^{-2} and 340 W m^{-2} at 200 nC, respectively.

Supplementary Note 5 | Total energy transfer efficiency under pulse output

V is the output voltage, Q is the corresponding transfer charge, and energy E is calculated by V and Q:

$$E = \int V dQ \tag{12}$$

For total energy transfer efficiency η_t of power management, Zi et al[1] calculates the η_t by the maximum output energy of power management over optimized energy of TENG on loads, following Niu's thought[2] which is calculated by the average power. With regard to pulse output, it has significant periodicity, thus, it is more accurate to calculate η_t by energy per cycle,

$$\eta_{t,p} = \frac{E_{out,max}}{E_{R,opt}} = \frac{\int V_{out,max} dQ_{out,max}}{\oint V_{R,opt} dQ_{R,opt}} \tag{13}$$

The $E_{R,opt}$ is the per cycle optimized output energy of TENG with loads directly connected, $V_{R,opt}$ and $Q_{R,opt}$ are the corresponding optimized voltage and charge. As shown in Fig. 3h-i, $E_{out,max}$ of FSCC power management system is 52.5 mJ m^{-2}, $E_{R,opt}$ of TENG is 69.4 mJ m^{-2}, so, $\eta_{t,p}$ is 75.6% calculated by equation 13.

Supplementary Note6. Total energy transfer efficiency under constant output

As for constant output, it is hard to measure output energy per cycle, hence, method used in pulse output is not adopted for constant output. Considering the constant characteristic, we used the method defined by Niu et al[2]. The total energy transformer efficiency is equal to the average output power $P_{out,avg}$ over the optimized average power $P_{R,opt,avg}$, and $P_{R,opt,avg}$ is the maximum average power of TENG with external loads directly connected. Herein, inspired by work of Xi et al[3], we employ the method of a rectifier and a filter to generate a constant output to measure $P_{out,avg}$ and $P_{R,opt,avg}$, as shown in Fig. 4a-b. In summary, total energy transfer efficiency of constant output is defined as follow:

$$\eta_{t,c} = \frac{P_{out,avg}}{P_{R,opt,avg}} = \frac{I_{out,avg} V_{out,avg}}{(V_{R,opt,avg})^2/R} \tag{14}$$

The maximum $P_{out,avg}$ of FSCC power management system is 37.09 mw m^{-2}, $P_{R,opt,avg}$ of TENG is 39.24 mw m^{-2} measured by stand circuit as shown in Fig. 4e, thus, the total energy transfer efficiency for FSCC power management is 94.5% calculated by Supplementary Equation 14.

Supplementary References:

1. Zi Y, *et al.* An inductor-free auto-power-management design built-in triboelectric nanogenerators. *Nano Energy* **31**, 302-310 (2017).

2. Niu S, Wang X, Yi F, Zhou YS, Wang ZL. A universal self-charging system driven by random biomechanical energy for sustainable operation of mobile electronics. *Nat Commun* **6**, 8975 (2015).

3. Xi F, *et al*. Universal power management strategy for triboelectric nanogenerator. *Nano Energy* **37**, 168-176 (2017).
