# Supplementary material for: Switched-capacitor-convertors based on fractal design for output power management of triboelectric nanogenerator
Source: Nat Commun. 2020 Apr 20;11:1883. doi: 10.1038/s41467-020-15373-y (PMC7171113; doi:10.1038/s41467-020-15373-y)
Supplement: Supplementary file 3 — Description of Additional Supplementary Files [file 41467_2020_15373_MOESM3_ESM.pdf]

Description of Additional Supplementary Files

File Name: Supplementary Movie 1
Description: The maximum output charge of TENG with FSCC power management.

File Name: Supplementary Movie 2
Description: Demonstration of lighting LEDs by FSCC power management with pulse output.

File Name: Supplementary Movie 3
Description: Demonstration of driving buzzer by FSCC power management with pulse output.

File Name: Supplementary Movie 4
Description: Driving digital vernier caliper continuously by FSCC power management with constant output.

File Name: Supplementary Movie 5
Description: Driving temperature hygrometer continuously by FSCC power management with constant output.
